# Counts: an outstanding challenge for log-ratio analysis of compositional data in the molecular biosciences

**DOI:** 10.1093/nargab/lqaa040

**Published:** 2020-06-19

**Authors:** David R Lovell, Xin-Yi Chua, Annette McGrath

**Affiliations:** Queensland University of Technology, Australia; Queensland University of Technology, Australia; Data61, Commonwealth Scientific and Industrial Research Organisation (CSIRO), Australia; Data61, Commonwealth Scientific and Industrial Research Organisation (CSIRO), Australia

## Abstract

Thanks to sequencing technology, modern molecular bioscience datasets are often compositions of counts, e.g. counts of amplicons, mRNAs, etc. While there is growing appreciation that compositional data need special analysis and interpretation, less well understood is the discrete nature of these count compositions (or, as we call them, lattice compositions) and the impact this has on statistical analysis, particularly log-ratio analysis (LRA) of pairwise association. While LRA methods are scale-invariant, count compositional data are not; consequently, the conclusions we draw from LRA of lattice compositions depend on the scale of counts involved. We know that additive variation affects the relative abundance of small counts more than large counts; here we show that additive (quantization) variation comes from the discrete nature of count data itself, as well as (biological) variation in the system under study and (technical) variation from measurement and analysis processes. Variation due to quantization is inevitable, but its impact on conclusions depends on the underlying scale and distribution of counts. We illustrate the different distributions of real molecular bioscience data from different experimental settings to show why it is vital to understand the distributional characteristics of count data before applying and drawing conclusions from compositional data analysis methods.

## INTRODUCTION

Compositional measurements are made in many molecular bioscience studies. At the beginning of the last decade, the implications of this for the analysis and interpretation of molecular bioscience data were not widely appreciated (1). By the end of the decade, this had changed dramatically with an increasing number of authors acknowledging how sampling and sequencing generally remove or distort information about the absolute abundance of components in omics data (2).

In tandem, methodologists from mathematics, statistics and computer science have been stimulated by the challenge of applying compositional philosophies and enhancing compositional techniques for bioscience data. Prominent methodologies include the log-ratio approach pioneered by Aitchison (3,4) and correspondence analysis (5); other methods have been suggested (6,7).

Currently, there is significant activity in the development, application and evaluation of new compositionally aware methods (as evidenced by this special issue). This is a fertile and exploratory era for methodological development; we are not yet at a stage where we have characterized the strengths and limitations of different approaches sufficiently well to know which methods are appropriate in different circumstances. Consistent with that, the intent of this paper is to deepen our understanding of one important aspect of compositional data analysis (CoDA) for bioscience data: the analysis and interpretation of counts.

Thanks especially to sequencing technology, molecular bioscience studies are replete with count data, or data derived from counts, e.g. counts of mRNA transcripts and counts of operational taxonomic units (OTUs) derived from assigning amplicon sequences to taxa in metagenomic surveys.

At first blush, this may seem unremarkable—after all, these counts form vectors of non-negative components and that is precisely the domain of CoDA (noting that the treatment of count zeros has long been recognized as a challenge for log-ratio analysis (LRA) (8)). However, as we will show, count compositional data (or as we term them *lattice compositions*) have some characteristics that could lead the unwary analyst astray, especially when exploring pairwise relationships between components.

We focus on LRA of lattice compositions because of the appeal and increasing popularity of this approach in molecular bioscience (2). We want to ensure that bioscientists (especially bioinformaticians) better understand the strengths and limitations of LRA and its applicability to different kinds of omics data, such as in *transcriptomics* where there tend to be fewer zeros and larger counts than in *metagenomics*, where data are often dominated by low and zero counts.

While this paper concentrates on LRA, it is important to be aware of other approaches that respect the underlying discrete nature of count compositional data, such as methods based on the log-normal Poisson distribution (9,10), correspondence analysis (5) (which can be related to LRA by the Box–Cox transformation), multinomial logistic-normal modeling (11–13), Dirichlet-multinomial models (14–16) and log-linear modeling with generalized estimating equations (17).

We also focus on LRA methods for assessing *pairwise association* between components because of their increasing use to explore interactions: ‘Inferring interactions among different microbial species within a community and understanding their influence on the environment is of central importance in ecology and medicine’ (18). This has resulted in the (inappropriate) use of correlation to construct networks of association between species (e.g. microbes, mRNAs) based on data about their relative abundance (e.g. see (19,20)). We stress that *‘*...in the absence of any other information or assumptions, correlation of relative abundances is just wrong’ (21); alternative approaches are being actively pursued (22,23).

The following sections aim to give all readers a deeper understanding of lattice compositions and their analysis by log-ratio methods. We begin with the geometry of the natural number lattice and its compositions and provide interactive graphics in the Supplementary Data to give intuition about concepts including closure, the simplex and coordinates. We also show how lattice compositions relate to fundamental concepts in number theory.

Next, we show how count data carry information about the *scale* (i.e. the relative extent and size) of counts and then explore the implications of this for LRA of pairwise association. We demonstrate that small counts can form a big part of bioscience datasets and discuss the implications of this for bioinformaticians and quantitative bioscientists who seek to draw sound conclusions from compositions of counts.

## THE GEOMETRY OF LATTICE COMPOSITIONS

We coin the term ‘natural number lattice compositions’ to describe compositions of count data. This emphasizes that the components of these data come from the set of natural numbers that we count with $\mathbb {N} = \lbrace 0, 1, 2, \dots \rbrace$, and that when we measure *D* components, they form a *D*-dimensional lattice $\mathrm{L}_{\mathbb {N}^D}$ that consists of points (*x*_1_, *x*_2_, …,*x*_*D*_), where $x \in \mathbb {N}$. *‘When we speak of lattice systems, we are imagining grids of points in space connected like monkey bars on a playground’* (24).

We appreciate that the term *‘*count composition’ is conventionally (and very understandably) used to describe compositions of counts. However, counts are not the only kind of compositional data that take discrete values: most empirical compositional data will be measured and recorded with finite precision and will therefore exist on a lattice. In using the term ‘lattice composition’ in this paper, we want to highlight the connection between these kinds of compositional data and the mathematical study of lattices and number theory so that deeper understanding might emerge, e.g. links to Euclid’s orchard and the Riemann zeta function discussed below.

In bioscience, the *components* or *parts* of a composition could be things like counts of different molecules, nucleotide sequences or OTUs. When we treat these data as compositions, we consider only the relative values of the components (i.e. their ratios) to be informative (3). Thus, the compositions (1, 2, 3) and (100, 200, 300) are compositionally equivalent, even though these two vectors of counts may carry different information, e.g. in the context of a species abundance study.

In the biosciences, lattice compositions arise in two ways: *experimentally*—when processes like sampling, sample preparation and sequencing remove information about the absolute abundance of components in the system being measured; and *mathematically*—typically when numbers are converted to proportions, percentages, ppm or ‘normalized’ by dividing through by some total, a process known as *closure* in CoDA. This conversion from absolute to relative abundances destroys information: given only relative abundance data, we cannot say how many species, transcripts, etc. were present in the original sample, just as knowing only the proportion of votes in an election tells us nothing about how many votes were actually cast. Less obviously, a range of familiar statistical methods (such as correlation (21)) are no longer applicable to relative data: this has been a driving motivation for CoDA.

**Figure 1.**
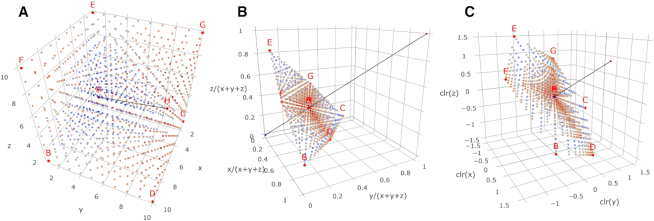
(**A**) The three-dimensional lattice of 1000 (*x*, *y*, $z$) triples where *x*, *y*, $z$ are integers from 1 to 10. Points are colored from blue to red as distance to the origin increases. The line from (1, 1, 1) to (10, 10, 10) is shown for reference, and the extreme points are labeled A–H. (**B**) The lattice of points in (A) after *closure*, i.e. dividing each component *x*, *y*, $z$ by the total *x* + *y* + $z$. The triangular section of the plane *x* + *y* + $z$ = 1 where the points lie on is called the *simplex* in three dimensions, $\mathcal {S}^3$. This is a many-to-one transformation because all points in (A) that lie on the same ray from the origin (i.e. (*kx*, *ky*, *kz*) for some positive *k*) will map to one point in $\mathcal {S}^3$, as is the case for points A and H in (A). Of the 1000 unique points in (A), there remain 841 unique points on this simplex. The line from (0, 0, 0) to (1, 1, 1) is shown for reference and the extreme points from (A) are labeled. (**C**) The lattice of points in (A) after the *centered log-ratio* (clr) transformation, i.e. dividing the log of each component log *x*, log *y*, log $z$ by the log of the geometric mean of all components log *g*_*m*_(*x*, *y*, $z$) = 1/3log *xyz*. These points lie on the plane log *x* + log *y* + log $z$ = 0. There is a one-to-one mapping between the points on the simplex in (B) and the points on this clr-plane. The line from (0, 0, 0) to (1, 1, 1) is shown for reference and the extreme points from (A) are labeled. (Interactive versions of these plots are available in the Supplementary Data.)

Figure 1A shows 1000 (*x*, *y*, $z$) triples on the natural number lattice and Figure 1B shows the corresponding lattice compositions formed by closure, i.e. dividing these triples by the total (*x* + *y* + $z$). It is important to appreciate that closure will project any point on the natural number of lattice onto the triangular *simplex*$\mathcal {S}^3$ in Figure 1B and, once transformed in this way, there is no return to the original (i.e. absolute) counts in $\mathbb {N}^3$ using the closed (i.e. relative) data alone.

Each point on the simplex corresponds to an equivalence class of vectors that lie on the ray from the origin through that point. For reference, Figure 1B shows the ray corresponding to the (*x*, *y*, $z$) triples where *x* = *y* = $z$. In other words, closure maps (infinitely) many vectors of *D*-dimensional counts to a single point in the *D*-dimensional simplex $\mathcal {S}^D$. But what about lattice points? How many of the 1000 points in Figure 1A will map to the same points in Figure 1B? Number theory, a branch of mathematics that studies the integers and integer-valued functions, tells us that if a lattice point is picked at random in *D* dimensions, the probability that it is visible from the origin is 1/ζ(*D*), where ζ(*D*) is the Riemann zeta function (25). This suggests there will be around 1000/ζ(3) ≈ 832 points visible from the origin in Figure 1B; there are actually 841 unique points in the closed data.

The triangular simplex $\mathcal {S}^3$ in Figure 1B is the basis of the *ternary diagram* used to display three quantities that sum to a constant; in essence a ternary diagram gives a 2D view of $\mathcal {S}^3$ as one would see from looking along the ray (1, 1, 1). LRA uses transformation to map the (constrained) simplex $\mathcal {S}^D$ to the (unconstrained) space of real numbers $\mathbb {R}^D$ in which statistical methods can be applied without fear of creating results that are not valid compositions. These transformations include the clr, *arithmetic log-ratio* (alr) and *isometric log-ratio* (ilr) transforms, as explained in (4). Once statistical analysis has been conducted in clr-, alr- or ilr-spaces, the results can be inversely transformed back to the corresponding simplex.

**Figure 2.**
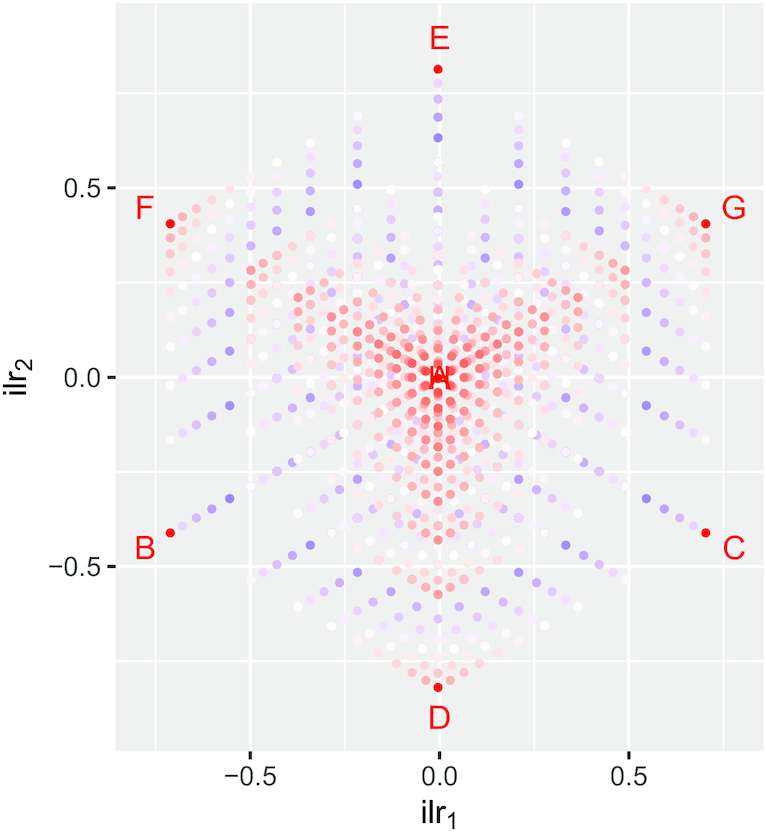
An isometric log-ratio transformation of the 1000 (*x*, *y*, $z$) triples in Figure 1A. Isometric log-ratios define a family of transformations rather than a single transform and we have used the specific ilr transformation $\mathrm{ilr}_1 = -\sqrt{1/2}\log _{10}(x/y), \mathrm{ilr}_2=-\sqrt{2/3}\log _{10}(\sqrt{xy}/z)$, which corresponds to the view of the clr-plane in Figure 1C along the ray (1,1,1). Points are colored and the extreme points are labeled as in Figure 1—note that corner points A and H map to the same point.

Figure 1C shows the 1000 (*x*, *y*, $z$) triples on the natural number lattice after clr-transformation onto the centered log-ratio plane in $\mathbb {R}^3$. As with closure, this is a many-to-one mapping and there are 841 unique points in the clr-transformed data, e.g. the corner points A (1, 1, 1) and H (10, 10, 10) in Figure 1A are mapped to (0, 0, 0) by clr-transformation, as is every other point where *x* = *y* = $z$. The perspective view in Figure 1C aims to show how the clr-transformation maps compositions to a plane in $\mathbb {R}^3$, specifically the plane where log *x* + log *y* + log $z$ = 0. Isometric log-ratio transformation into *ilr-coordinates* (26) provides a (*D* − 1)-dimensional view of compositions of *D* parts, and we will use that approach to help visualize three-part compositions in this paper (Figure 2).

Bioscience experiments can easily yield compositions with hundreds or thousands of components: why are we considering only three? By using ratios of components, LRA is *subcompositionally coherent*: *‘*measures of association or measures of dissimilarity between components...are unaffected by considering subcompositions’ (27). This means that we can explore the simplest case of pairwise association (say between components *x* and *y*) in the presence of just one more component ($z$) knowing that our conclusions about *x* and *y* would be the same even if there were many more components present. This enables us to visualize key aspects of LRA in two and three dimensions.

The aims of this visual presentation of lattice compositions and their transformations are (i) to give readers a geometric intuition of LRA and (ii) to show how the discrete nature of lattice compositions manifests in the simplex, clr- and ilr-coordinates. Now we are well prepared to consider what information lattice compositions carry into the simplex.

## COUNT DATA CARRIES SCALE INFORMATION

*Scale invariance* is a fundamental principle of CoDA. Put simply, scale invariance means that the functions we use to analyze compositions return identical results when we scale compositions by multiplying all components by a constant. ‘...two compositions x and X are regarded as equivalent ...if there is some a > 0 such that X = ax. ...it follows that any meaningful function of a composition must satisfy the requirement of scale invariance f(ax) = f(x)’ (28).

Count compositional data (and therefore lattice compositions) are *not* scale-invariant representations of continuous data. In general, scaled-down lattice compositions cannot be exactly represented on the lattice. For example, scaling down the lattice composition (86, 75, 309) by factor of 10 gives the composition (8.6, 7.5, 30.9), which is not on the natural number lattice; the nearest lattice composition approximation is (9, 8, 31). The original lattice composition carries more information than its scaled-down approximation. The impact of this *quantization* is felt most at small scales: *‘Consider the metric spaces*$\mathbb {Z}_n$*and*$\mathbb {R}_n$*. Their small-scale structure—their topology—is entirely different, but on the large scale they resemble each other closely’* (29). This situation is analogous to display resolution on digital devices: IBM’s 1981 Color Graphics Adaptor (CGA) had 320 × 200 pixels; full high definition displays have 1920 × 1080; both can display images, but the CGA device’s approximation is obvious.

**Figure 3.**
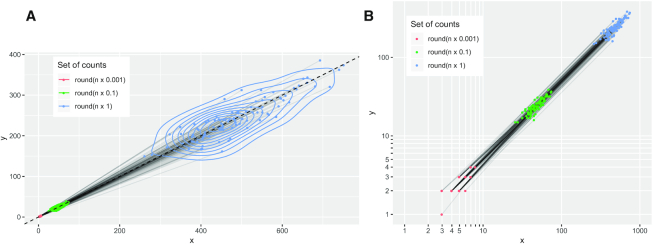
(**A**) Imagine an environment populated by species *x* and *y*. We take 100 samples and find that there are about 500 of species *x* in each sample and that there are around half as many of species *y* in each sample (blue points). To simulate what we might have seen if these species were 10-fold less abundant, we divide all our counts by 10 and round them to the nearest whole number (green points). (This does not simulate sampling variation, but our intent is to illustrate the impact of quantization on counts.) We repeat this process to simulate 100-fold reductions in abundance (red points). (**B**) As our counts decrease by orders of magnitude, we see (on a log-scale) that the natural number lattice affords a coarser representation of the initial counts.

We illustrate the impact of quantization in Figure 3 by taking a set of counts (*x*, *y*, $z$) and repeatedly scaling them down by a factor of 10, and then then rounding them to the nearest integer. Initially, there were 100 unique samples; after scaling counts down by 100-fold, there are only nine distinct count pairs on the lattice. Clearly, low counts cannot carry as much information as high counts, and in the next section, we explore the consequences that this has on measures of pairwise association.

## PAIRWISE ASSOCIATION IN LATTICE COMPOSITIONS

The statistical assessment of pairwise association is a key method in making sense of bioscience data. It needs to be approached with particular care for compositional data because change in one component necessarily affects the relative abundance of others. The parts of a composition are correlated by design and are not free to vary independently: an increase in the proportion of one component demands a decrease in at least one other. This is one way of understanding why Pearson’s correlation is not a valid measure of association in compositions, as Pearson himself showed in 1897 (30).

*Proportionality* is a valid measure of association for data that carry relative information (21). In this section, we consider three related statistics that measure the extent to which pairs of components are proportional, and look at their behavior on lattice compositions.

To introduce these statistics, we first define $\mathbf {X}$ as an *N* × *D* matrix of *N* observations where the *i*th observation is the *D*-part composition $\mathbf {x}_i = (x_{i1}, \dots , x_{ij}, \dots , x_{iD})$. The clr representation of composition $\mathbf {x}_i$ is the logarithm of the components after dividing by the geometric mean of $\mathbf {x}_i$:

$$\begin{equation*} \mathrm{clr}(\mathbf {x}_i)=\left(\log \frac{x_{i1}}{\mathrm{g_{m}(\mathbf {x}_i)}},\dots ,\log \frac{x_{ij}}{\mathrm{g_{m}(\mathbf {x}_i)}},\dots ,\log \frac{x_{iD}}{\mathrm{g_{m}(\mathbf {x}_i)}}\right). \end{equation*}$$

Hence, the sum of the elements of $\mathrm{clr}(\mathbf {x}_i)$ is zero. The row-wise clr-transformed version of $\mathbf {X}$ is written as $\mathbf {C}$, its *j*th column is written as $\mathbf {C}_{\cdot j}$ and its element *i*, *j* is denoted by *cij*.

The first proportionality statistic is the *variance of the log-ratios* of parts *j* and *k* (3):

(1) $$\begin{eqnarray*} \mathrm{vlr}(\mathbf {X}_{\cdot j}, \mathbf {X}_{\cdot k}) &\triangleq& \underset{i}{\mathrm{var}} \left(\log \frac{x_{ij}}{x_{ik}}\right) \nonumber \\ &=& \underset{i}{\mathrm{var}} (\log x_{ij} - \log x_{ik}) \nonumber \\ &=& \underset{i}{\mathrm{var}} ( c_{ij} - c_{ik}). \end{eqnarray*}$$

(This is also referred to as ‘log-ratio variance’; however ‘variance of log-ratios’ makes the order of operations clear.) When $\mathbf {X}_{\cdot j}$ and $\mathbf {X}_{\cdot k}$ are exactly proportional, the variance of their log-ratios is 0. However, this statistic has been criticized because, when pairs of components are not exactly proportional, ‘it is hard to interpret as it lacks a scale. That is, it is unclear what constitutes a large or small value...(does a value of 0.1 indicate strong dependence, weak dependence or no dependence?)’ (31). This led to the proposal of scaled versions of vlr, introduced in (21) and developed further in (32):

(2) $$\begin{eqnarray*} \phi _{\rm s}(\mathbf {C}_{\cdot j}, \mathbf {C}_{\cdot k}) \triangleq \frac{\mathrm{var}_i (c_{ij} - c_{ik})}{\mathrm{var}_i (c_{ij} + c_{ik})}, \end{eqnarray*}$$

(3) $$\begin{eqnarray*} \rho _{\rm p}(\mathbf {C}_{\cdot j}, \mathbf {C}_{\cdot k}) \triangleq \frac{1-\phi _s(\mathbf {C}_{\cdot j}, \mathbf {C}_{\cdot k})}{1+\phi _s(\mathbf {C}_{\cdot j}, \mathbf {C}_{\cdot k})}. \end{eqnarray*}$$

Like vlr, ϕ_*s*_ is zero when its arguments are exactly proportional and positive otherwise, so it could be thought of as a distance from proportionality. ρ_*p*_ maps this distance from [0, ∞) to the interval [1, −1), reminiscent of Pearson’s correlation coefficient. Note that Equation (2) is not defined for compositions with two parts since, by definition, *c*_*i*1_ + *c*_*i*2_ = 0.

Each of these statistics has strengths and limitations (32) and development of new statistics to measure pairwise association continues (33). However, the behavior of vlr, ϕ_*s*_ and ρ_*p*_ with lattice compositions has not yet been explored.

**Figure 4.**
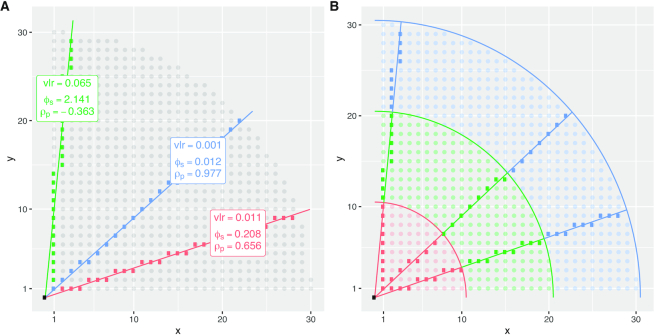
Lattice compositions cannot, in general, exactly represent proportional relationships between pairs of components, and this affects statistical measures of proportionality. (**A**) This plot shows three different colored lines of exact proportionality between *x* and *y*; the colored points show their discrete lattice approximation. The slopes of the lines are 1/3 (red), 9/10 (blue) and 10/1 (green). The corresponding proportionality statistics of the lattice approximations are shown in the boxes using logarithms to base 10. Note that we have used a third component $z$ = 1 (not shown) to ensure that ϕ_*s*_ and ρ_*p*_ are defined. Also, we have defined the lattice approximation so that 1 is the minimum *x* and *y* value to avoid taking logarithms of 0. (**B**) Keeping the same lines of exact proportionality as in (A), we now use colors to indicate different regions of the natural number lattice from radius 1–10 (red), 10–20 (green) and 20–30 (blue). By sweeping a line of proportionality through positive slopes from 0 to ∞ (i.e. angles 0–90° to the *x*-axis), we can record the values of vlr, ϕ_*s*_ and ρ_*p*_ in different regions of the lattice approximation.

Figure 4A shows that lattice compositions cannot exactly represent proportional relationships (*x* = *ky*) between pairs of components, except for equality i.e. *k* = 1. Consequently, the statistics vlr, ϕ_*s*_ and ρ_*p*_ do not indicate precise proportionality when applied to the lattice approximation. Instead, vlr and ϕ_*s*_ are >0 and ρ_*p*_ is <1 (in one case, <0) even though the underlying continuous relationships that generated the lattice compositions are perfectly proportional. To evaluate how these proportionality statistics behave across a range of lattice approximations we systematically sampled lines of positive slopes between zero and infinity (Figure 4B). Since real count data will have a finite range, we evaluated vlr, ϕ_*s*_ and ρ_*p*_ on finite ranges of lattice points as indicated by the colored annular in Figure 4B.

**Figure 5.**
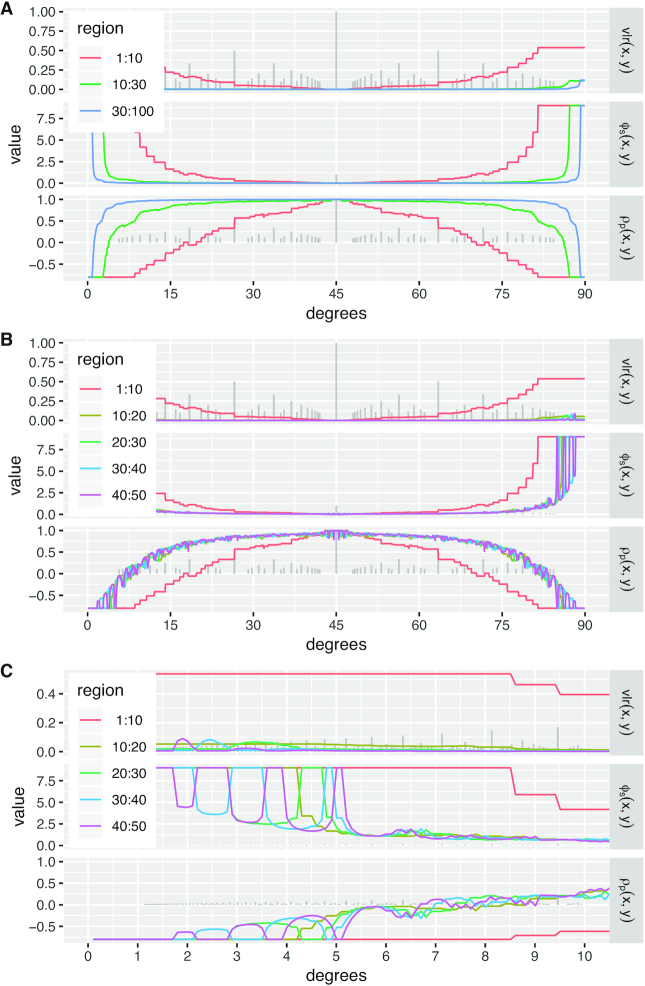
These plots show the behavior of the proportionality statistics vlr, ϕ_*s*_ and ρ_*p*_ for lattice approximations to a line of proportionality as it is swept through positive slopes from 0 to ∞ as described in Figure 4B. Different colored lines indicate these statistics reported on different regions of the natural number lattice, e.g. from radius 1–10 (red), 10–20 (green) and 30–100 (blue) in (A). For reference, we plot a sample of Euclid’s orchard (50) in the background out to radius 50 to show the finite number of rational slopes available to lattice points. As in Figure 4A, we use logarithms to base 10 and have defined the lattice approximation so that 1 is the minimum *x* and *y* value to avoid taking logarithms of 0. (**A**) As the region of lattice available to approximate the line of proportionality increases, the proportionality statistics move closer to the values they would have for continuous compositions, i.e. vlr = ϕ_*s*_ = 0 and ρ_*p*_ = 1. (**B**) When counts span smaller regions, in this case regions of around 10 points on the lattice, the proportionality statistics vary markedly at angles close to horizontal and vertical, corresponding to low count values in either *x* or *y*. (**C**) Close-up of the curves from (B) for angles 0–10°. In theory, these curves should be piecewise constant with discontinuities rather than the connected steps shown. This reflects our method of generating these curves by sampling the statistics at regular intervals.

Figure 5 shows some of the curves we observed, demonstrating clearly that measures of pairwise proportionality behave differently, sometimes very differently, on lattice compositions from $\mathrm{L}_{\mathbb {N}^3}$ than their continuous counterparts. (vlr and ϕ_*s*_ would be 0 and ρ_*p*_ would be 1 in all panels of Figure 5 for proportional compositions from $\mathbb {R}^3_+$.)

In theory, the curves in Figure 5 are piecewise constant, with discontinuities as the lattice approximations involve different points. Our plots connect values of vlr, ϕ_*s*_ and ρ_*p*_ sampled at 900 equiangular intervals from (0 + δ)° to (90 − δ)° for a small value of δ.

Figure 5A shows that the lattice approximation of lines of continuous slope improves as we increase the radius of lattice points involved; radius 1 : 10 (the red curve) shows the impact of quantization on proportionality most markedly. Figure 5B shows more clearly that it is not just the slope of the line that effects the lattice approximation, but the *scale* of the counts involved. Figure 5C shows that different regions of the lattice exhibit quantization effects at different angles.

Before we discuss the implications of these findings, we need to address one count value that we have so far carefully avoided: zero.

## ZEROS IN LATTICE COMPOSITIONS

Modeling and analysis of zeros in count data has received a lot of attention (34,35), especially in LRA of compositions (8,36–37) since zero is the natural enemy of the logarithm. Lattice compositions can have zeros and it is important to appreciate that these can arise for different reasons that in turn, demand different treatments and interpretation (37,38).

Treatment of count zeros is a rich topic in its own right and we are not going to pursue it in detail here. Instead, our aim is to look briefly at the results of popular methods for zero replacement in the context of LRA.

**Figure 6.**
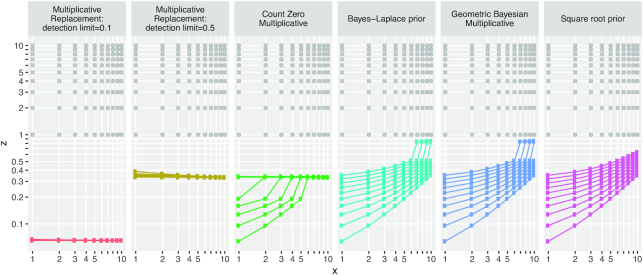
Five strategies for zero replacement (8) using the zCompositions package (36) applied to the three-part lattice compositions formed by the 1100 (*x*, *y*, $z$) triples where *x* and *y* are integers from 1 to 10, and $z$ is an integer from 0 to 10. Each panel shows the lattice of counts for *x* and $z$ with no zeros (the gray points) along with the value used to replace $z$ = 0 (the colored points). Colored points connected by lines have the same *y* value (from 1 to 10). While all the gray points lie on the natural number lattice $\mathrm{L}_{\mathbb {N}^2}$, none of the zero replacements lie on the lattice of next lower magnitude $\mathrm{L}_{0.1\mathbb {N}^2}$.

Figure 6 shows the results of five methods for zero replacement implemented in the zCompositions package (36). From these plots, we can observe that there appear to be two main families of zero imputation: multiplicative and Bayesian, with count zero multiplicative (CZM) replacement somewhat of a hybrid. Choice of detection limit in multiplicative replacement clearly affects how close the imputed values are to the compositions on the lattice. This is also an issue in CZM replacement. As Martín-Fernández *et al.* note, ‘this treatment adds spurious correlation between rare parts resulting from adding a fixed value, shared by the parts with count zeros’ (8).

With respect to lattice compositions, clearly none of the methods replace zeros with values that necessarily lie on the lattice of next lower magnitude to $\mathrm{L}_\mathbb {N}$, i.e. the lattice of ‘tenths’ $\mathrm{L}_{0.1\mathbb {N}}$. We can envisage ‘lattice-friendly’ zero-replacement methods whose results belong to {0.1, 0.2, …, 0.9}.

## SMALL COUNTS CAN BE A BIG ISSUE

We have shown that count data carry scale information into the scale-invariant machinery of LRA. We have also shown that when counts are low, and when the range of counts is limited, pairwise measures of compositional association (i.e. proportionality) can be very different in lattice compositions than their continuous counterparts. The question now is does this matter in practice? And if so, when?

The answer depends on the scale of the counts involved, and in this section, we consider the scale and distribution of some real datasets.

**Figure 7.**
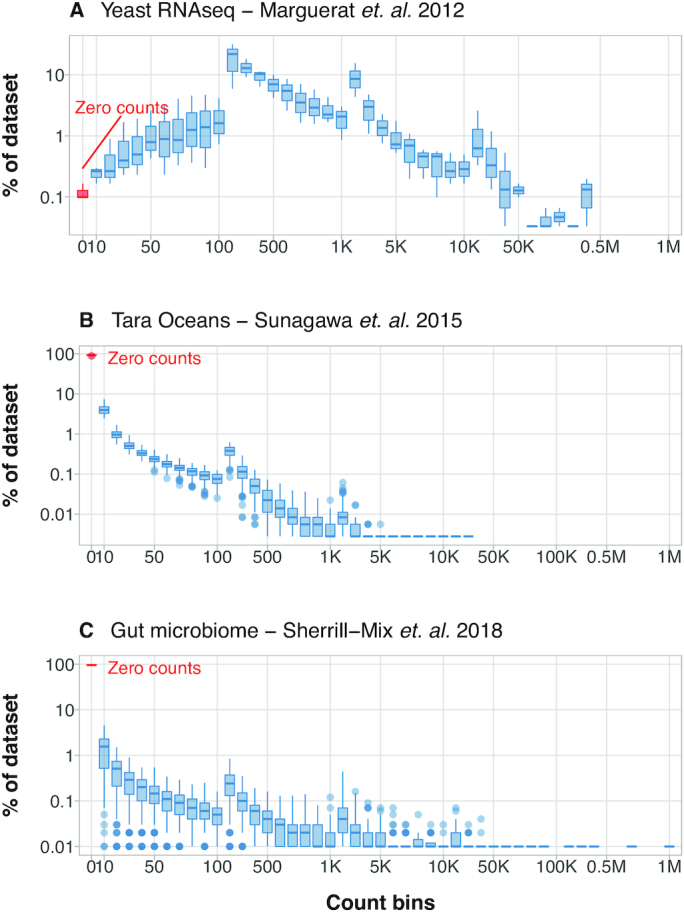
The distribution of counts from three molecular bioscience studies that make use of high-throughput sequencing. The *x*-axes show count bins using a log-like binning method (see text for description) while the proportion (in %) of the data are on the *y*-axis. There are two distinct shapes in the datasets, especially when comparing the proportion of zero counts (labeled in red) against the remainder of the dataset. RNA-seq data (**A**) do not consist of many zeros, whereas environmental and metagenomic studies (**B**) and (**C**) are inundated with zero counts. The plots on the right are a close-up of the shaded gray region on the left.

Figure 7 summarizes the count distribution for three kinds of sequencing-based molecular bioscience studies:

**Yeast RNA-seq** data (39) are from a study to better understand cell-regulatory functions in cell proliferation and quiescence, comparing yeast transcript expression levels over *N* = 16 time points.
  Approximately 38M reads, 2.4M reads per sample.
**Tara ocean** data (40) examined the biodiversity catalog of marine micro-organisms (prokaryotes) using shot-gun sequencing and environmental data from the Tara Oceans expedition collected over *N* = 139 stations.
  Approximately 14M reads, 0.102M reads per sample.
**Gut microbiome** study (41) aimed to test the Hubell’s neutral model of ecology by showing that bacterial diversity in gut samples was positively associated with animal mass. The study was performed over *N* = 265 individuals across 10 classes of animals, representing 64 species, from very small body mass (e.g. bedbugs, flies, bees) to very large body mass (e.g. sharks and whales).
  Approximately 17M reads, 0.065M reads per sample.

The dynamic range of these count data is challenging to present and Figure 7 shows counts on the *x*-axis using ‘logarithmic-like’ bins. The bins includes zero counts, and accumulate counts in the groups [0, 1–10, 11–20, ..., 101–200, 201–300, ..., 1001–2000, ...] with each bin width increasing by a factor of 10 when reaching the next order. The *y*-axis shows the percentage of the dataset contained in each bin. As each study consists of multiple samples (16, 139 and 265, respectively), a boxplot summarizes the range per bin, revealing the count variability between samples within a study.

These studies fall into two groups, with the zero count boxplot highlighted and labeled in red in the graph. The RNA-seq dataset has far fewer zero counts than the two metagenomic studies in which zeros account for up 90% of the data. To show the trend of the remaining counts, the right-hand side of the figures shows a close-up of the grayed region on the left-hand side. The microbiome studies are ‘cataloging’ experiments aimed to survey organisms across a broad and diverse landscape. The landscape for the RNA-seq experiment is quite narrow by comparison: we expect to see similar mRNAs present in each sample.

Abundance distributions in microbial community studies display a ‘long tail’ of low-abundance organisms (42). This tail is referred to as the ‘rare biosphere’ and often accounts for the vast majority of the phylogenetic diversity present. The rare biosphere has most commonly been defined using relative abundance of <0.1 or 0.01% (42). Despite their rarity, these low-abundance taxa have been shown to perform essential roles in biochemical processes, community assembly and stability and resilience (43). For example, *Desulfosporosinus* spp., representing only 0.006% of the total community, play a pivotal role in sulfate reduction and carbon flow in peatland soils (44).

A common approach in metagenomic and environmental DNA (eDNA) processing is to perform low-abundance filtering of OTUs since these could be a result of technical variations in the library preparation and sequencing. ‘It has been shown that when unique reads, such as chimeras and singletons, are withheld [i.e., retained] in analysis, the estimation of diversity can be severely inflated (45)’. Unfortunately there is no consensus about the best filtering standard, and methods vary from study to study. Many studies will remove singleton OTUs (those that only appear once in the entire study) (46,47), otherwise the filtering is generally based on either a minimum count (typically 10 for a conservative approach) or at a minimum relative abundance (e.g. 0.001) (48). The choice of filtering method depends on the biological question.

**Figure 8.**
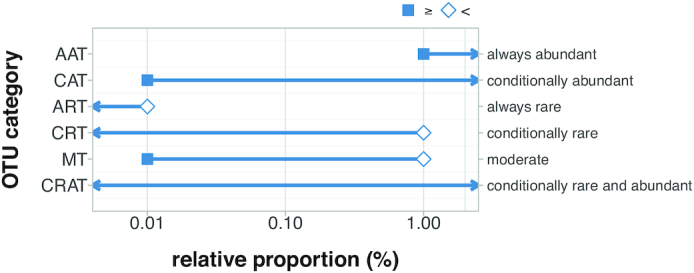
A graphical representation of the six OTU categories (*y*-axis) defined in (46). The relative abundance range for each category is shown on the *x*-axis, with solid squares representing ≥*x* and the open diamonds representing <*x*.

Xue *et al.* describe six categories of OTUs from abundant to rare (46) (see Table 1 and Figure 8). Using these definitions, we filter our three case study datasets and report the results in Tables 2 and 3. Using the six OTU categories proposed by Xue *et al.*, the differences between the three case study datasets stand out most clearly in Table 3.

**Yeast RNA-seq**: while the bulk of mRNAs (41%) are conditional rare (CRT), they only make up 16% of the sequenced reads, compared to half the reads being from *moderate* features (MT).
**Tara Oceans**: there are no *always abundant taxa* (AAT) at all. Nearly 80% of the OTUs are tagged as *always rare taxa* (ART), but these are mainly zero data points. The *conditionally rare* (CRT) OTUs make up 75% of the sequenced reads, which makes sense as the samples are collected across 139 stations around the globe.
**Gut microbiome**: there are no *always abundant* (AAT), *conditionally abundant* (CAT) or even *moderate* OTUs. This indicates that the data are very sparse. The majority of the data are within the *conditionally rare and abundant* taxa (CRAT), indicating that OTUs are very specific to different environments.

**Table 1.** The six categories of OTUs from abundant to rare as defined in (46); see also Figure 8

Category	Relative abundance (%)	No. of samples
AAT: always abundant taxa	≥1	all
CAT: conditionally abundant taxa	≥0.01	all
	≥1	some
ART: always rare taxa	<0.01	all
CRT: conditionally rare taxa	<0.01 − 1	all
	<1	all
MT: moderate taxa	0.01 − 1	all
CRAT: conditionally rare and abundant taxa	<0.01 − ≥1	any?

**Table 2.** The number (and proportions) of remaining (a) sequences, (b) features (mRNA in the case of RNA-seq data and OTUs in the case of metagenomic data), (c) data with zero counts and (d) data counts between 2 and 9, after different filtering methods for the three example studies

		(a)		(b)		(c)		(d)	
Dataset	Threshold	No. of sequences	%	No. of features	%	No. of zeros	%	No. [2-9]	%
**Yeast RNAseq**, *N*= 16 samples				(mRNA)					
	No filtering	37 710 728	100.00	3034	100.00	56	100.00	278	100.00
	Relative abundance ≥ .0001	34 330 805	91.04	2019	66.55	0	0.00	7	2.52
	Relative abundance ≥ .001	22 464 080	59.57	317	10.45	0	0.00	0	0.00
	Relative abundance ≥ .01	8 277 104	21.95	24	0.79	0	0.00	0	0.00
	Count ≥ 2	37 710 696	100.00	3031	99.90	8	14.29	278	100.00
	Count ≥ 10	37 708 896	100.00	3029	99.84	7	12.50	269	96.76
**Tara Oceans**, *N*= 139 samples				(OTU)					
	No filtering	14 129 941	100.00	35 651	100.00	4 394 814	100.00	199 424	100.00
	Relative abundance ≥ .0001	13 093 797	92.67	7250	20.34	595 938	13.56	155 003	77.73
	reRelative abundance ≥ .001	8 241 812	58.33	2450	6.87	135 678	3.09	56 849	28.51
	Relative abundance ≥ .01	1 499 364	10.61	113	0.32	5324	0.12	2369	1.19
	Count ≥ 2	13 941 637	98.67	19 803	55.55	2 222 449	50.57	199 424	100.00
	Count ≥ 10	13 147 108	93.04	7483	20.99	623 333	14.18	157 107	78.78
**Gut microbiome**, *N*= 265 samples				(OTU)					
	No filtering	17 365 964	100.00	10 000	100.00	2 535 419	100.00	37 964	100.00
	Relative abundance ≥ .0001	17 266 878	99.43	9862	98.62	2 499 064	98.57	37 893	99.81
	Relative abundance ≥ .001	16 302 087	93.87	8992	89.92	2 276 347	89.78	34 302	90.35
	Relative abundance ≥ .01	12 125 721	69.82	1521	15.21	370 082	14.60	7431	19.57
	Count ≥ 2	17 346 927	99.89	9897	98.97	2 508 181	98.93	37 964	100.00
	Count ≥ 10	17 180 567	98.93	9419	94.19	2 382 756	93.98	37 141	97.83

The first row shows no filtering of the dataset, so for yeast, there are 37.7M sequences, of which 56 are zero counts and 278 have counts between 2 and 9; these sequences collapse down to 3K features after clustering. The second row shows in the Tara Oceans dataset that by filtering on relative abundance ≥0.0001, we reduce the number of OTUs from 35 651 down to 7250 (20%), which is comparable to using the threshold of absolute minimum count of 10. The number of zero count data has also reduced significantly from 4.4M to 596K.

**Table 3.** The number (and proportions) of remaining (a) sequences, (b) features (mRNA in the case of RNAseq data and OTUs in the case of metgenomic data), (c) data with zero counts and (d) data counts between 2 and 9, after categorizing the features (mRNA or OTUs) into the size groups as defined by (46); see also Table 1 and Figure 8

		(a)		(b)		(c)		(d)	
Dataset	Code	No. of sequences	%	No. of features	%	No. of zeros	%	No. [2–9]	%
**Yeast RNA-seq**, *N*= 16 samples				(mRNA)					
	AAT	3 888 103	10.31	3	0.10				
	CAT	6 844 187	18.15	19	0.63				
	ART	1 696 865	4.50	1015	33.45	56	100	271	97.48
	CRT	5 980 056	15.86	1253	41.30			7	2.52
	MT	19 079 234	50.59	742	24.46				
	CRAT	222 283	0.59	2	0.07				
**Tara Oceans**, *N* = 139 samples				(OTU)					
	AAT								
	CAT	779 811	5.52	2	0.01	NA	NA	1	0.00
	ART	239 961	1.70	28 401	79.66	3 798 876	86.44	44 421	22.27
	CRT	10 615 556	75.13	7121	19.97	590 614	13.44	152 632	76.54
	MT	462 899	3.28	16	0.04	NA	NA	2	0.00
	CRAT	2 031 714	14.38	111	0.31	5324	0.12	2368	1.19
**Gut microbiome**, N=265 samples				(OTU)					
	AAT								
	CAT								
	ART	1245	0.01	138	1.38	36 355	1.43	71	0.19
	CRT	3 063 513	17.64	8341	83.41	2 128 982	83.97	30 462	80.24
	MT								
	CRAT	14 301 206	82.35	1521	15.21	370 082	14.60	7431	19.57

AAT: always abundant taxa; ART: always rare taxa; CAT: conditionally abundant taxa; CRAT: conditionally rare and abundant taxa; CRT: conditionally rare taxa; MT: moderate taxa.

It is clear that low and small counts are very prevalent in biological datasets, particularly in microbiome studies where the OTUs that they represent account for the majority of the biodiversity and play important roles in microbial communities. Awareness of the limitations of analytic techniques and their implications for small counts is essential to drawing appropriate conclusions from these datasets.

## DISCUSSION AND CONCLUSION

As quantitative bioscientists, it is critical we have a clear view of all the sources of variation in our data. This is so that we understand the extent to which a numerical representation of the system under study reflects the *biological variation* of interest, compared to all the *technical variation* we have introduced in our attempts to measure that system. It is also critical that we understand the methods we might apply to analyze and interpret these numbers so that we can be confident that our conclusions and findings are about the biology of interest and not artifacts of our analysis methods.

There is growing appreciation of the compositional nature of many molecular bioscience datasets (49) and a natural desire to apply CoDA methods that have a strong mathematical basis and proved useful in other domains (3). However, we must be careful to respect the true nature of the data we apply these methods to. In this paper, we have focused intently on the underlying discrete nature of count compositions and shown how it introduces quantization variation that can eclipse the biological variation of interest, especially when counts are low.

Even though LRA is scale-invariant, applying it to scale-dependent data such as counts means that the conclusions we draw from this analysis of lattice compositions depend on the scale of the data we apply it to. We presented three examples of real bioscience data from different experimental settings to illustrate the variety of scales and count distributions that can arise in the molecular biosciences.

For bioinformaticians and quantitative bioscientists, this is a salutary reminder that the tools we use to analyze our data produce results regardless of whether the tools are appropriate for the data; our wisdom (i.e. the knowledge of how to use knowledge) lies in appreciating the strengths and limitations of different analytical techniques in different situations. When it comes to assessing pairwise association between counts of components in a lattice composition, we must beware of situations where we will be unable to observe the components of interest in sufficient number to resolve a proportional relationship amidst the variation due to quantization.

While this presents an outstanding challenge to bioinformaticians and data analysts, it may be a challenge better taken up by experimentalists. If we could find ways to systematically deplete high-abundance molecular species (as can be done with ribosomal RNAs) to increase the counts of less abundant members, LRA of these adjusted compositions could be more confidently applied to explore relationships between rarer species.

In summary, while technology enables us to read many millions of nucleotide sequences, the diversity of sequences present in different experimental settings can give rise to big numbers of small (and zero) counts. As we have shown, lattice compositions have fundamental limits to the amount of information they can represent and these limits become apparent when counts are low. LRA approaches to measuring pairwise association will struggle in this setting. The implications of this are that counts remain an outstanding challenge for LRA of compositional data in the molecular bioscience, especially metagenomic data.

## Supplementary Material

lqaa040_Supplemental_File
