# Supplementary material for: Counts: an outstanding challenge for log-ratio analysis of compositional data in the molecular biosciences
Source: NAR Genom Bioinform. 2020 Jun 19;2(2):lqaa040. doi: 10.1093/nargab/lqaa040 (PMC7671413; doi:10.1093/nargab/lqaa040)
Supplement: lqaa040_Supplemental_File [file lqaa040_supplemental_file.html]

Counts: An outstanding challenge for compositional data analysis


# Counts: An outstanding challenge for compositional data analysis

### *Supplementary Information*

#### *David Lovell, Xin-Yi Chua, Annette McGrath*

#### *29 February 2020*

# About this Supplementary Information

This HTML Supplementary Information provides interactive versions of Figures 1(a-c) of *Counts: an outstanding challenge for compositional data analysis in the molecular biosciences*.

## Make a \(10\times 10\times 10\) cube of counts

Here we create a 3-dimensional lattice of 1000 \((x,y,z)\) triples where \(x\), \(y\), \(z\) are integers from 1 to 10 with the extreme points labeled A–H. We also include some vectors that we will use for reference.

```
rbind(
  expand.grid(x=1:10,    y=1:10,    z=1:10)    %>% mutate(example="ref: 10x10x10",   ID=NA),
  expand.grid(x=c(1,10), y=c(1,10), z=c(1,10)) %>% mutate(example="extremes",        ID=LETTERS[row_number()]),  
  tibble     (x=c(0,1),  y=c(0,1),  z=c(0,1))  %>% mutate(example="vector(1,1,1)",   ID=NA),
  tibble     (x=c(1,10), y=c(1,10), z=c(1,10)) %>% mutate(example="vector(10,10,10)",ID=NA)
) %>% as.tbl() -> counts
```

For each triple of counts \((x, y, z)\) we add further columns such as

- the *closure* \((x, y, z)/(x+y+z)\)
- the *centred log-ratio* \((\log x, \log y, \log z)/\log(\sqrt[3]{xyz})\)
- the *isometric logratio projection* of the count triple onto \((\sqrt{1/2}\log(x/y), -\sqrt{2/3}\log(\sqrt{xy}/z))\)

### 3D plot of raw counts

Show the set of lattice points in Euclidean space

### 3D plot of closed counts on the simplex

### 3D plot of clr transformed counts
